# Supplementary material for: Identification of photoperiod-induced specific miRNAs in the adrenal glands of Sunite sheep (Ovis aries)
Source: Front Vet Sci. 2022 Jul 22;9:888207. doi: 10.3389/fvets.2022.888207 (PMC9354845; doi:10.3389/fvets.2022.888207)
Supplement: Supplementary file 1 [file Data_Sheet_1.ZIP › Supplementary file/miRDeep2 file.zip!/miRDeep2 file/Novel_Structure/LP42A1/Novel_236.pdf]

Provisional ID	:	NC_019458.2_501
Score total	:	1.5
Score for star read(s)	:	-1.3
Score for read counts	:	0
Score for mfe	:	1.2
Score for randfold	:	1.6
Score for cons. seed	:	
Total read count	:	103
Mature read count	:	103
Loop read count	:	0
Star read count	:	0

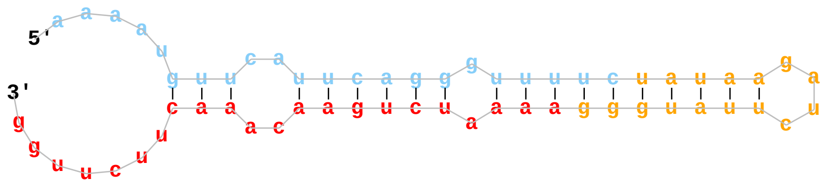

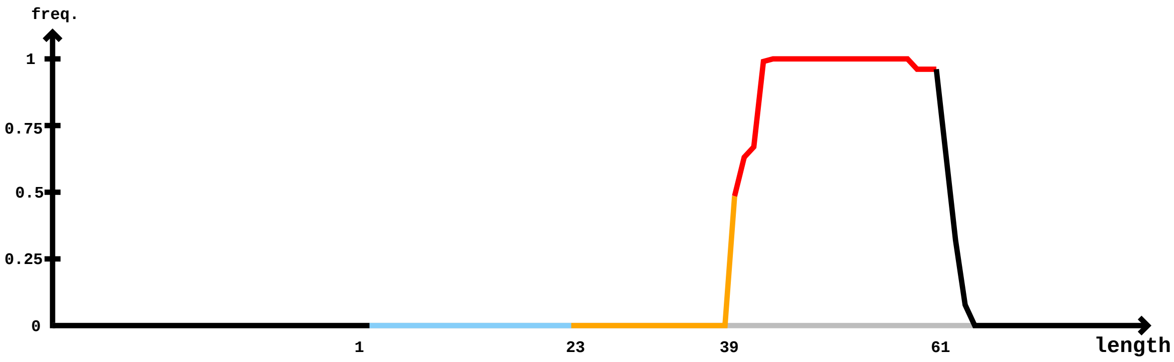

Star Mature

```
5'- uuucuguaaguucaauauuauuggguaggcuaaaauguucauucaggguuuucuauaagaucuuaugggaaaaucugaacaaacuucuuggccaacccaauccuuccaaaua   -3'    exp
    ...................(((((((.((((((...(((..((((((.((((((((((....)))))))))).))))))..)))...)))))).))))))).........   reads    mm    sample
    .....................................................................aaaaucugaacaaacuuUu........................   4        1     seq
    .....................................................................aaaaucugaacaaacuuUuugg.....................   45       1     seq
    .....................................................................aaaaucugaacaaacuuUuuggc....................   1        1     seq
    ......................................................................aaaucugaacaaacuuUuugg.....................   14       1     seq
    ......................................................................aaaucugaacaaacuucuuggc....................   1        0     seq
    .......................................................................aaucugaacaaacuuUuuggc....................   4        1     seq
    ........................................................................aucugaacaaacuuUuuggcc...................   25       1     seq
    ........................................................................aucugaacaaacuuUuuggcca..................   8        1     seq
    .........................................................................ucugaacaaacuuUuuggc....................   1        1     seq
```
